# Longitudinal Analysis of T and B Cell Receptor Repertoire Transcripts Reveal Dynamic Immune Response in COVID-19 Patients

**DOI:** 10.3389/fimmu.2020.582010

**Published:** 2020-09-30

**Authors:** Xuefeng Niu, Song Li, Pingchao Li, Wenjing Pan, Qian Wang, Ying Feng, Xiaoneng Mo, Qihong Yan, Xianmiao Ye, Jia Luo, Linbing Qu, Daniel Weber, Miranda L. Byrne-Steele, Zhe Wang, Fengjia Yu, Fang Li, Richard M. Myers, Michael T. Lotze, Nanshan Zhong, Jian Han, Ling Chen

**Affiliations:** ^1^State Key Laboratory of Respiratory Disease, National Clinical Research Center for Respiratory Disease, Guangzhou Institute of Respiratory Health, The First Affiliated Hospital of Guangzhou Medical University, Guangzhou, China; ^2^Jiangsu Industrial Technology Research Institute (JITRI), Applied Adaptome Immunology Institute, Nanjing, China; ^3^iRepertoire Inc., Huntsville, AL, United States; ^4^Guangzhou Regenerative Medicine and Health-Guangdong Laboratory (GRMH-GDL), Guangdong Laboratory of Computational Biomedicine, Guangzhou Institutes of Biomedicine and Health, Chinese Academy of Sciences, Guangzhou, China; ^5^HudsonAlpha Institute for Biotechnology, Huntsville, AL, United States; ^6^Guangzhou Eighth People’s Hospital, Guangzhou Medical University, Guangzhou, China; ^7^Department of Surgery, University of Pittsburgh Medical Center, Pittsburgh, PA, United States

**Keywords:** COVID-19, SARS-CoV-2, T cell receptor, B cell receptor, immune repertoire, biomarker

## Abstract

Severe COVID-19 is associated with profound lymphopenia and an elevated neutrophil to lymphocyte ratio. We applied a novel dimer avoidance multiplexed polymerase chain reaction next-generation sequencing assay to analyze T (TCR) and B cell receptor (BCR) repertoires. Surprisingly, TCR repertoires were markedly diminished during the early onset of severe disease but recovered during the convalescent stage. Monitoring TCR repertoires could serve as an indicative biomarker to predict disease progression and recovery. Panoramic concurrent assessment of BCR repertoires demonstrated isotype switching and a transient but dramatic early IgA expansion. Dominant B cell clonal expansion with decreased diversity occurred following recovery from infection. Profound changes in T cell homeostasis raise critical questions about the early events in COVID-19 infection and demonstrate that immune repertoire analysis is a promising method for evaluating emergent host immunity to SARS-CoV-2 viral infection, with great implications for assessing vaccination and other immunological therapies.

## Introduction

The current outbreak of coronavirus disease (COVID-19) was first reported in Wuhan, China (1, 2). The virus, severe acute respiratory syndrome coronavirus 2 (SARS-CoV-2), is a virus closely related to SARS-CoV, endemic in 2003 (3). The virus caused low respiratory tract pneumonia, but it also affects multiple organs such as the kidney, liver, brain, gastrointestinal tract, and heart. The virus spreads by respiratory droplets, urine, and feces (4, 5). Clinical symptoms of SARS-CoV-2 include fever, cough, shortness of breath, and chest pain. The pneumonia in those with this disease is characterized by bilateral ground-glass opacities identified on chest CT scans (4, 5). The majority of COVID-19 patients show mild or moderate symptoms and recover after proper clinical care. However, some COVID-19 patients rapidly develop severe pneumonia, subsequent multi-organ failure, and death (6). Pathologic examination reveals diffuse alveolar damage, proteinaceous plugs, and a prominent myeloid infiltrate and a paucity of lymphocytes (7–10).

Despite global pandemic threats of COVID-19 disease, the host immune response against SARS-CoV-2 infection remains poorly understood. Lymphopenia is common in SARS-CoV-2 infected patients and was found as well as in SARS-CoV and Middle East respiratory syndrome (MERS) patients (4, 6, 11). It has been observed that the counts of total T cells, CD4+ and CD8+ subtype T cells, were dramatically reduced in severe COVID-19 cases with increased expression of programmed death-ligand 1 (PD-1) and T cell immunoglobulin mucin 3 (Tim-3), indicating activation and T cell exhaustion (12). Monitoring the dynamics of lymphocyte number and phenotype has been suggested as a means to predict the severity of COVID-19 (13). Older COVID-19 patients with comorbidities are at a particularly high risk of severe pneumonia and death. The diminished T cell repertoire and progressive defects in T cell and B cell function in older patients could limit viral clearance and prolong the innate proinflammatory response (14, 15).

The human adaptive immune system consists of both naïve and memory cells, which express either cell surface B cell receptors (BCRs) or T cells receptors (TCRs), in aggregate termed the adaptome (16). Recently, next-generation sequencing (NGS) of BCRs and TCRs have been used widely to evaluate immunity (17–21). Analyzing the full repertoires could provide a better understanding of the immune response to SARS-CoV-2 and other infections. Because all seven of the immune repertoire chains, including IgH (all isotypes), IgK, IgL, TCR-Alpha, Beta chains, and TCR – Gamma, Delta chains are amplified under the same conditions in one PCR reaction with our method, the expression level of these genes can be directly compared with an inclusive and quantitative pattern (16). Here, we deeply investigated the peripheral blood repertoire from patients throughout their course of COVID-19 disease, demonstrating dynamic changes over the disease course.

## Materials and Methods

### Isolation of PBMCs and RNA Extraction

Six-eight milliliter PBMCs were isolated by density gradient separation on a Ficoll-Hypaque gradient as previously described (22) (GE Healthcare, Chicago, IL, USA). Initially 2 million cells were used to extract total RNAs using TRIzol™ LS reagent according to the manufacturer’s protocol Invitrogen.

### Unbiased Amplification of TCRs and BCRs

In this study, iR-RepSeq-plus 7-Chain Cassette (iRepertoire, catalog no. iR+7chain-HLRI-C) was used to generate NGS libraries covering all TCR and BCR chains including TCR-beta, -alpha, -delta, -gamma, and BCR-IgH, -IgK, -IgL. All seven chains were amplified in a single assay using a strategy which allows the incorporation of unique molecular identifiers (UMIs) during the reverse transcription (RT) step. One disposable cassette is for one sample’s library preparation; all necessary reagents for amplification and purification are preloaded into the cassette. Extracted RNA (1000 ng) with an appropriate volume of RT mix and nuclease-free water were added into the cassette, which was processed by the iR-Processor. The instrument can automatically set up and carry out all amplification and purification. Briefly, RT is performed using Qiagen OneStep RT-PCR mix (Qiagen). First-strand cDNA was selected, and remnant primers were removed by SPRIselect bead selection (Beckman Coulter) followed by the second round of binding and extension with the V-gene primer mix. After binding and extension, SPRIselect beads were used to purify the first and second strand synthesis products. Library amplification is performed with a pair of primers that are specific for communal sites engineered onto the 5’ end of the C- and V- primers used in first and second-strand synthesis, the detailed information would be found on iRepertoire Inc website. The final constructed library includes Illumina dual index sequencing adapters, a 10-nucleotide UMIs, and an 8-nucleotide internal barcode associated with the C-gene primer. Amplified libraries were multiplexed and pooled for sequencing on the Illumina NovaSeq platform with a 500-cycle kit (250 paired-end reads) through a commercial sequencing service lab (Personal Biotechnology Co., Ltd, Shanghai, China). The output of the immune receptor sequence covers within the first framework region through the beginning of the constant region including CDR1, CDR2, and CDR3.

### Data Collection and Bioinformatics Analysis

Raw data were analyzed using the previously described iRmap program (17, 23). Briefly, sequence reads were de-multiplexed according to barcode sequences at the 5’ end of reads from the constant region. Reads were then trimmed according to their base qualities with a 2-base sliding window. If either quality value in this window is lower than 20, this sequence stretches from the window to 3’ end was trimmed out from the original read. Trimmed pair-end reads were joined together through overlapping alignment with a modified Needleman-Wunsch algorithm. If paired forward and reverse reads in the overlapping region were not perfectly matched, both forward and reverse reads were thrown out without further consideration. The merged reads were mapped using a Smith-Waterman algorithm to germline V, D, J, and C reference sequences downloaded from the IMGT website (http://www.imgt.org/vquest/refseqh.html#VQUEST) (24). To define the CDR3 region, the position of CDR3 boundaries of reference sequences from the IMGT database was migrated onto reads through mapping results, and the resulting CDR3 regions were extracted and translated into amino acids. The dataset was condensed by the combination of UMIs and CDR3 regions to remove incorrect CDR3s introduced by sequencing and amplification. Reads with the same combination of CDR3 and UMI were condensed into one UMI count. The effect of percentage of TCRs and BCRs was analyzed by an unpaired two-tailed *t-*test. All the tests were implemented in the GraphPad Prism 7.0 software (GraphPad Software, La Jolla, California, USA).

## Results

### Dynamic Repertoire Changes Over the Disease Course of COVID-19

We collected 23 peripheral blood mononuclear cells (PBMCs) samples from COVID-19 patients. Among them, three patients each provided four samples at times that ranged from early stage to recovery stage (**Supplementary Figure 1**). The cohort includes 4 males and 6 females, with a median age of 57 years old (ranging from 33 to 81 years) (**Supplementary Table 1** and **Supplementary Figure 1**). Peripheral blood samples from 15 Asian healthy donors were collected as normal controls, which included 9 males and 6 females, with ages ranging from 22 to 67 years (**Supplementary Table 1**). The inclusion criteria for healthy donors were: 1) no apparent chronic disease; and 2) no diagnosis of acute diseases in the past three months. We used four sequential PBMC samples obtained from a 59-year-old healthy seasonal influenza vaccinee as a longitudinal healthy control. We amplified the immune repertoires including all TCR chains (TCR-alpha, TRA; TCR-beta, TRB; TCR-delta, TRD; TCR-gamma, TRG) and BCR chains (IgH, including the various IgH isotypes; IgK; and IgL) in one PCR reaction in an unbiased way, based on a strategy of using the first set of primer pairs for each V-J to allow extension with tags that enabled a second set of primers to be utilized for global amplification of all seven chains. Each sample was allotted approximately 5 million sequencing reads (**Supplementary Table 2**). The data were further analyzed by iRepertoire’s data analysis pipeline (17, 23).

**Figure 1:**
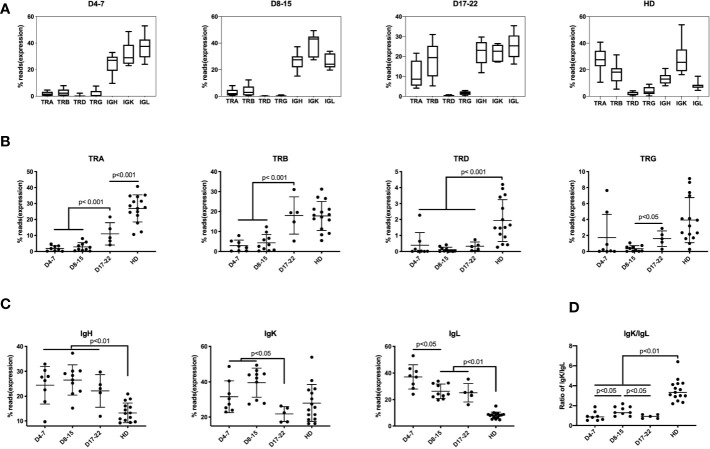
Immune repertoires of TRA, TRB, TRD, TRG, and IgH, IgK, IgL in PBMCs of COVID-19 patients at different disease stages. The proportion of reads for each immune chain are presented as mean value ± SD in the boxplots. The box extends from the 25th to 75th percentiles. The whiskers go down to the smallest value and up to the largest value. The samples from COVID-19 patients are divided into three groups based on time points after COVID-19 infection: **(A)** Samples of 4–7 days (D4-7) following symptom onset (n = 8); Samples of 8-15 days (D8-15) following symptom onset (n = 10); Samples of 17-22 days (D17-22) following symptom onset (n = 5); Samples of Asian healthy donors (HD) (n = 15). **(B)** The proportion of reads of TRA, TRB, TRD and TRG at three groups were compared along with HD group. **(C)** The proportion of IGH, IGK, and IGL reads at three groups were compared along with HD group. **(D)** The ratio of IgK/IgL at three groups were compared along with HD group. The difference in proportion values was assessed by an unpaired two-tailed *t-*test.

Previously, lymphopenia was observed in COVID-19 patients, with especially diminished T lymphocytes, but the expression of individual TCRs and BCRs was not determined (4, 5). Six of 21 samples (28.57%) found mild to moderate lymphopenia (define as below 1 × 10^9^ L) (**Supplementary Table 3**). We found that unique TCR reads (n = 23) was significantly lower than that of healthy donors (HD) (n = 15) (**Figure 1A**). Compared with health donors, the average percentage of TRA reads in the immune repertoire was reduced from 28.3% to 4.9%, and that of TRB reads was reduced from 18.7% to 5.2% (decreases of 5.8 times and 3.6 times, respectively). The proportions of IgH, IgK and IgL in COVID-19 samples were higher than that in healthy donors (**Figure 1A**), especially the increase of IgL from 8.5% to 23.5%.

We divided these samples into groups according to the course of the disease: D4-7 group, samples from 4 to 7 days after symptom onset (n = 8); D8–15 group, samples from 8 to 15 days after symptom onset (n = 10); and D17–22 group, samples from 17 to 22 days after symptom onset (n = 5), which represents as disease recovery for the time points the patients were SARS-CoV-2 virus-negative and the samples were collected before discharge. The analysis demonstrates low frequency of mRNA expression of the TRA and TRB chains in samples in D4–7 group and D8–15 group. In contrast, expression of these TCR chains increases on Day17–22 group (convalescent stage). Surprisingly, TRB reads in D17–22 group is similar to that of the normal control group which showed no significant difference between D17–22 and HD group, but the proportion of D17–22 group showed significantly higher than D4–7 group and D8–15 group (p < 0.001). This demonstrates that the TRB expression can be used as a reference index for recovery from SARS-COV-2 viral infection. TRA, TRD, and TRG percentage of D17–22 groups were significantly lower than the HD group (**Figure 1B**), suggesting dynamics changes of different TCRs following SARS-CoV-2 infection. We notice that percentage of TCRs and BCRs share nearly half of reads in the repertoire of HD group. In contrast the inhibition of TCRs in COVID-19 infection, the IgH and IgL proportion showed rapidly increased as early as day 3 onset of infection (D4–7 group, **Figure 1C**), and maintained as long as 22 days after infection. The IgK showed much slower increasing on D4–7 group and peaked on D8–15 group, then quickly dropped on D17–22 group. These aberrant IgK/IgL ratio had been found in tumor BCR, which indicated selection events in tumor and other B cell related disease (25). This indicated selection events in COVID-19 infection especially on days 8–15 onset of syndrome. Further studies are needed to elaborate the mechanism of this finding. Taken together, TCR expression was markedly reduced in the early disease stage, and IgH/IgK/IgL expression increased, likely as the result of the humoral immune response to the viral infection.

### BCR Repertoire Analysis Demonstrated an Early IgM to IgG Isotype Switch and a Transient IgA Surge Following Disease Onset

**Figure 2:**
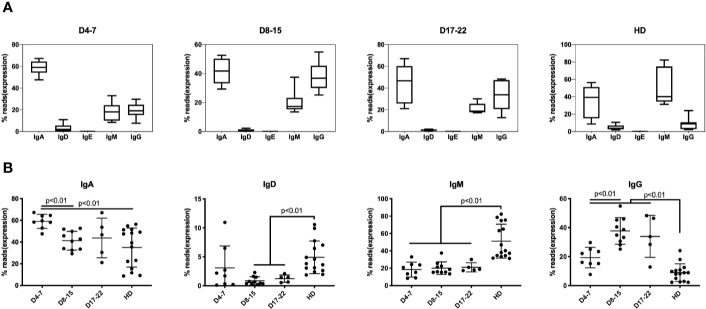
Immunoglobulin isotypes in BCR repertoires in PBMCs of COVID-19 patients at individual disease stages. The proportion of IgA, IgG, IgD, IgE, and IgM isotype expressing B cells are presented as mean ± SD in the boxplots. The box extends from the 25th to 75th percentiles. The whiskers go down to the smallest value and up to the largest value. **(A)** The proportion of IgA, IgD, IgE, IgM, and IgG in three groups based on time points following COVID-19 infection along with HD group. **(B)** The proportion of IgA, IgD, IgM, and IgG was compared at three groups along with HD group. The difference in proportion values was assessed by an unpaired two-tailed *t-*test.

In response to SARS-COV-2 infection, IgM expressing B cells are first mobilized with T cell mediated class-switch to the IgG isotype. The median percentage of IgM-expressing B cells is 19.5% in samples (n = 8) on days 4–7 of COVID-19 patients and maintained lower level in all COVID-19 infection, which showed significantly lower than HD group (43.6%) (**Figure 2A**). The IgG expression subsequently increased about 10% in the early stages of infection (D4–7 group) as compared to the HD group, increasing to nearly 40% of total IgH expression on D8–15 group, and IgH percentage on all stages showed significantly higher than did of HD group (**Figure 2B**). Another notable change that we observed consistently was that the IgA proportion during early infection (days 4–7) increased significantly to 60.1% as compared to 42.6% in the HD group, and significantly decreasing to 43.3% on D8–15 group (**Figures 2A, B**).

The transient IgA surge at early stage of infection mRNA expression and IgA-plasmablasts expansion was detected but much late of serum level (26). These results indicate that IgM/IgG/IgA are rapidly mobilized in response to coronavirus infection. Previous studies showed that such mobilization prevented viral-induced pathology in the upper respiratory tract of mice infected with influenza (27).

### Dynamic Changes in the Repertoires Enable a Holistic View of COVID-19 Infection and Enable Close Monitoring of Immunity During Disease Progression

**Figure 3:**
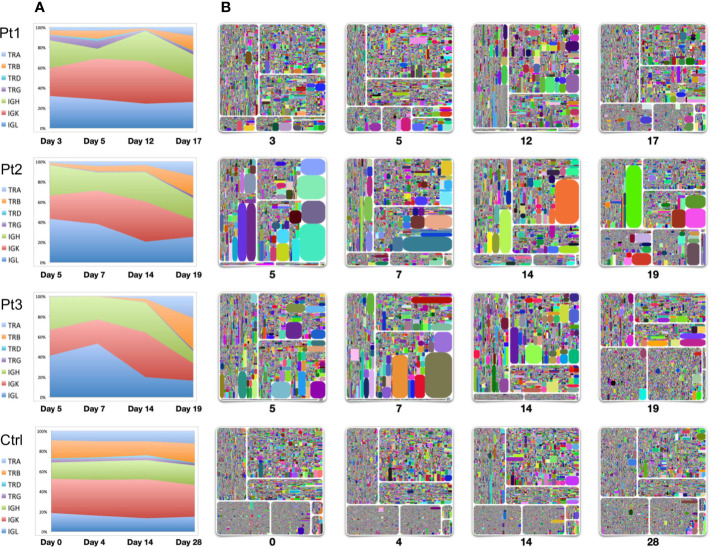
Longitudinal analysis of TCR and BCR repertoires of four individuals. Samples were collected from three patients from early-mid stages after symptom onset (days 3, 5, 7, 12, 14) to the convalescent stage (days 17, 19). Samples from a healthy seasonal quadrivalent influenza vaccinee before and after vaccination (days 0, 4, 14, and 28) were used as a control. **(A)** The proportion of TCR-TRA, -TRB, -TRD, -TRG and BCR-IgH, -IgK, -IgL in the adaptive immune reservoirs. **(B)** Treemap plots representing unique CDR3 clonotypes in each of the seven chains on the indicated time points after symptom onset. The seven treemap plots each represent TCR-beta, -alpha, -delta, -gamma, and BCR-IgH, -IgK, -IgL. A rectangle in a treemap plot represents a unique clonotype. The size of a rectangle denotes the relative frequency of an individual CDR3 sequence, and the varying square size reflects areas of clonal expansion within the immune repertoire sampled. The color of the individual CDR3 sequence in each Treemap plot was randomly chosen, and thus, the colors do not precisely match between individual plots. From the left upper plot clockwise to the bottom plots: IgH, IgK, IgL, TRB, TRA, TRD, and TRG.

To evaluate the dynamics of immune repertoire changes in COVID-19 patients, we longitudinally tracked the immune repertoires of three patients at four time points collected from early onset to the convalescent stage. We found that TRB was extremely low in the early disease stage. TRB gradually increased with the improvement throughout the disease, especially during convalescence. The percentage of TRB expression based on the number of reads increased to the same level as that observed in healthy controls (**Figures 1**, **3**). This result suggests that measures of TRB return to normal number of reads is a critical and readily available reference indicator of recovery. We also found that the IgH expression within the repertoires gradually reduced as the disease diminished. Thus, it was the highest on the days 4–7 after symptom onset and returned to normal levels (~20%) on the 19th or 20th day (**Figure 3**). The proportion of IgL expression was highest on days 4–7 following symptom onset with decreases during the course of the disease (Pt2 and Pt3). Compared with the repertoires of a healthy seasonal influenza vaccinee, who received influenza vaccine yearly, the proportions of TCR and BCR chains were relatively stable before vaccination (day 0) and following vaccination over 28 days (**Figure 3**).

Longitudinal analysis of these three patients using treemap analysis that determines the relative abundance of individual clonotypes showed that TRB expression returned to normal during the convalescent stage. Except for the presence of some dominant clones in the TRB expression profile, the diversity and frequency of individual clonotypes was significantly improved by the time of convalescence (time point 4). A significant feature of IgH/IgK/IgL expression is the presence of dominant clonal expansion around two weeks after infection (the third time point, day 12 for Pt1, day 14 for Pt2 and Pt3), suggesting that selection events are drastically for BCRs on this stage. Studies on these clonally expanded IgH sequences may use a source for developing neutralizing antibodies.

**Figure 4:**
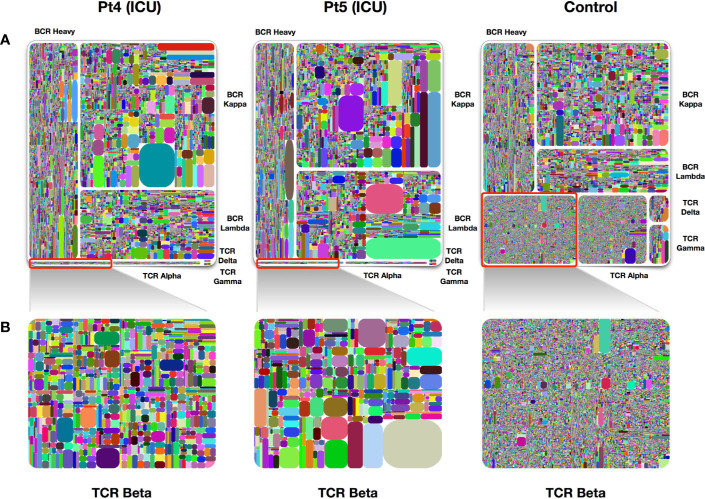
Treemap plots of TCR and BCR repertoires in PBMCs of ICU patients. **(A)** Treemap plots of the seven individual adaptive immune chains from patient 4 and patient 5 on day 4 following symptoms onsets. The control treemap plot was from the same healthy person before vaccination with a seasonal quadrivalent influenza vaccine. **(B)** Treemap plots containing total TRB unique CDR3 clonotypes, each represents patients 4, patient 5, and a healthy control. A rectangle in a treemap plot represents a unique clonotype. The size of a rectangle denotes the relative frequency of an individual CDR3 sequence. The color of the individual CDR3 sequence in each Treemap plot was randomly chosen, and thus, the colors do not match between the plots.

Approximately 15% of COVID-19 patients develop severe disease (4). We analyzed two samples obtained at day 4 and day 6 after symptom onset from two patients who were admitted to the intensive care unit (ICU) due to the severity of their disease. Similar to what we found in other samples of early infection, IgH/IgK/IgL became largely dominant in the treemap plots, whereas TRB expression was dramatically reduced as compared to healthy individuals (**Figure 4**). Many dominant clones were found on examination of IgK expression and the diversity of this chain was lower than that in healthy control individuals.

## Discussion

Studies of TCR and BCR dynamics in COVID-19 patients provide valuable insights into the natural history of the disease, the host response, and providing insights enabling assessment of effective clinical treatment. Here, we demonstrate that the evolving immune system’s response to SARS-COV-2 infection involves clearly delineated patterns during early infection through convalescence. The recent availability of the seven chain DAM-PCR/NGS to assess dynamic changes in individual TCRs and BCRs allowed visualization of the full repertoires, demonstrating how adaptive immunity and disease progress through to recovery. The abnormal expression profile of TCRs and BCRs during early disease stage, in particular, TRA and TRB expression, and the recovery of this expression during the convalescence stage, is available as a reference indicator of disease recovery. The initial absence of TRA and TRB expression expands and elucidates previously available clinical data demonstrating lymphopenia in COVID-19 patients. It implies that T cells are either undergoing acute apoptotic events or, alternatively, leave the circulation. Examination of individuals early in infection or at autopsy suggests that there is limited traffic of T cells to the lungs. Examination of lymphoid organs early during infection has not been reported. T cells play an important role in virus clearance and the subsequent establishment of antibody-mediated protection against virus infection (28, 29). T cell-deficient mice but not B-cell-deficient mice with MERS-CoV infection resulted in persistence of the virus in the lung, indicating the direct role of T cells to clear this virus (29). More recently, tissue resident innate lymphoid cells (ILC2s) have been demonstrated to be sufficient to promote IgA switch in viral-responsive B cells (30). The decreased CD8+ T cells in COVID-19 patients associated with markers of activation and exhaustion is an early indicator for requiring ICU treatment (12). Consistent with prior evidence that the lowest lymphocytes count was on day 4 following symptom onset (6), we found that the expression of TCRs dramatically decreased during the early stage of infection (days 4–7) of patients in both moderate and severe cases. One possibility is that virus-induced epithelial destruction and resultant pneumonia increased vascular permeability and chemokine recruited T cells nonselective leak out into the lung (31). Further studies are needed to clarify if T cells are present in bronchoalveolar lavage fluid from the lungs of severe patients and in lungs of people with mild infections in the early stage of infection.

In our measurements of the repertoire, we found that IgM/IgG and IgA B cell responses were mobilized early in response to viral infection. Clonal expansion of the IgH chain was evident after two weeks onset of symptoms. Although we cannot recognize antigen-specific clones, the clone expansion would find on tree-map of longitudinal samples (**Figure 3**). This implies the human adaptive immune system could mounts a vigorous early B cell response to this novel pathogenic virus. IgM-expressing B cells are believed to be the first cell type to expand following exposure to antigen. Measurement of SARS-CoV-2-specific IgM antibody can help rapidly diagnose viral infection (32). Expanded transcript levels of IgM and IgG expressing-B cells could be found as early as day 4 in SARS-CoV-2 infection in this study. Published studies demonstrate with serologic assessment expanded titers of IgM/IgG appear by days 7–14 following onset (33–35). Relative IgG expression levels can be even higher following virus clearance, increasing progressively for more than six months. This indicates that virus-specific antibodies are under persistent affinity maturation within the germinal center (22, 36, 37). Another surprising finding to us was the predominance of B cells expressing IgA very early following infection. To our knowledge, this is the first report that IgA-expressing B cells can be rapidly activated and be detectable in the peripheral blood upon infection with a novel virus. To understand how such early B cell-expressing IgA, and presumably IgA antibodies, are activated and increased in the peripheral blood is potentially bypassing normal T cell controls and utilizing ILCs within the lung to drive the switch (30). Isolation of IgA-specific antibodies may be important for early diagnosis and therapy. IgA plays an important role in mucosal immunity (38). The increasing percentage of IgA expressing B cells indicates that IgA may be synthesized and migrate widely to the respiratory tract, the gastrointestinal tract, or other mucosal sites to play an early immune function to clear virus (39).

Previous studies focused on the TCR-beta or IgH chain repertoires individually to evaluate the adaptive immune response to cancer immunotherapy, autoimmune disease, or viral infection (22, 37, 40–42). The multiplex dimer avoided multiplex-PCR (dam-PCR)/NGS method used here allows for inclusive and quantitative amplification of the TCR-alpha, -beta, -gamma, -delta chains and BCR-IgH, -IgK, -IgL simultaneously in one PCR reaction (16). To our knowledge, this is the first report to systemically elucidate the BCR and TCR repertoires of COVID-19 patients and should have broad applicability in assessing emergent vaccines and therapies for this and other viral and bacterial diseases.

Longitudinal analysis of B cells expressing each of the individual IgH, Igκ and Igλ chains can provide a holistic view of the antibody repertoire changes over time. Single-cell PCR of SARS-CoV-2-specific B cells combined with BCR repertoire analysis could accelerate the identification and cloning of neutralizing antibodies by NGS and classical antibody cloning (22, 43). By employing an integrated analysis of TCRs and BCRs, we gave a panorama view of repertoire dynamics after COVID-19 infection, but repertoires of each patients before infection were not available. The samples were obtained from 6-8 mL peripheral blood samples, which only represented a small fraction of the total blood in the circulation, so some clonotypes may be not included in the repertoire. SARS-CoV-2 specific repertoires of TCR and BCR were not examined, further studies should expand the repertoire dynamics of T/B cell subsets and give insight why lymphopenia occurs. One limitation of present study is that the dynamics of frequency of TCRs/BCRs could not be validated by lymphocytes number counting, C-Reaction Protein value, or sera titers. Integrated of repertoire sequencing and single-cell transcriptomics, antibody cloning may give comprehensive explanation of adaptive immune response against virus infection (44).

Taken together, the results of this study provide a much more detailed view of the immune dynamics of COVID-19 patients. Global and longitudinal analyses of adaptive immunity in COVID-19 patients could provide insights onto the mechanisms of virus infection, providing information for assessment during clinical treatment and assisting in the development of antiviral therapeutics and vaccines.

## Data Availability Statement

The datasets presented in this study can be found in online repositories. The names of the repository/repositories and accession number(s) can be found below: https://bigd.big.ac.cn/, PRJCA003775.

## Ethics Statement

The studies involving human participants were reviewed and approved by Ethical Review Committee of Guangzhou Medical University. The patients/participants provided their written informed consent to participate in this study.

## Author Contributions

LC, JH, and XN initiated and coordinated the project. LC and XN designed the experiments. XM and FL recruited the patients. PL and SL conducted the experiments. WP and DW performed the bioinformatics analysis. LC, JH, XN, WP, and SL analyzed the data. LC and XN wrote the manuscript. All authors contributed to the article and approved the submitted version.

## Funding

This work was supported by the National Natural Science Foundation of China (82041014 and 81661148056) and the special program for SARS-COV-2 research of Guangzhou Institute of Respiratory Health (GIRH) for LC and XN. China Postdoctoral Science Foundation (Grant No. 2020T130115ZX) for PL.

## Conflict of Interest

WP, SL, DW, MB-S, and JH are employees of iRepertoire, Inc.

The remaining authors declare that the research was conducted in the absence of any commercial or financial relationships that could be construed as a potential conflict of interest.
