# Corrigendum: Longitudinal Analysis of T and B Cell Receptor Repertoire Transcripts Reveal Dynamic Immune Response in COVID-19 Patients

**DOI:** 10.3389/fimmu.2020.633815

**Published:** 2020-12-21

**Authors:** Xuefeng Niu, Song Li, Pingchao Li, Wenjing Pan, Qian Wang, Ying Feng, Xiaoneng Mo, Qihong Yan, Xianmiao Ye, Jia Luo, Linbing Qu, Daniel Weber, Miranda L. Byrne-Steele, Zhe Wang, Fengjia Yu, Fang Li, Richard M. Myers, Michael T. Lotze, Nanshan Zhong, Jian Han, Ling Chen

**Affiliations:** ^1^ State Key Laboratory of Respiratory Disease, National Clinical Research Center for Respiratory Disease, Guangzhou Institute of Respiratory Health, The First Affiliated Hospital of Guangzhou Medical University, Guangzhou, China; ^2^ Jiangsu Industrial Technology Research Institute (JITRI), Applied Adaptome Immunology Institute, Nanjing, China; ^3^ iRepertoire Inc., Huntsville, AL, United States; ^4^ Guangzhou Regenerative Medicine and Health-Guangdong Laboratory (GRMH-GDL), Guangdong Laboratory of Computational Biomedicine, Guangzhou Institutes of Biomedicine and Health, Chinese Academy of Sciences, Guangzhou, China; ^5^ HudsonAlpha Institute for Biotechnology, Huntsville, AL, United States; ^6^ Guangzhou Eighth People’s Hospital, Guangzhou Medical University, Guangzhou, China; ^7^ Department of Surgery, University of Pittsburgh Medical Center, Pittsburgh, PA, United States

**Keywords:** COVID-19, SARS-CoV-2, T cell receptor, B cell receptor, immune repertoire, biomarker

In the original article, there was an error in the Data Availability Statement. The correct data accession number is PRJCA003775.

The authors apologize for this error and state that this does not change the scientific conclusions of the article in any way. The original article has been updated.

