# Supplementary figures and images for: Longitudinal Analysis of T and B Cell Receptor Repertoire Transcripts Reveal Dynamic Immune Response in COVID-19 Patients
Source: Front Immunol. 2020 Sep 30;11:582010. doi: 10.3389/fimmu.2020.582010 (PMC7561365; doi:10.3389/fimmu.2020.582010)

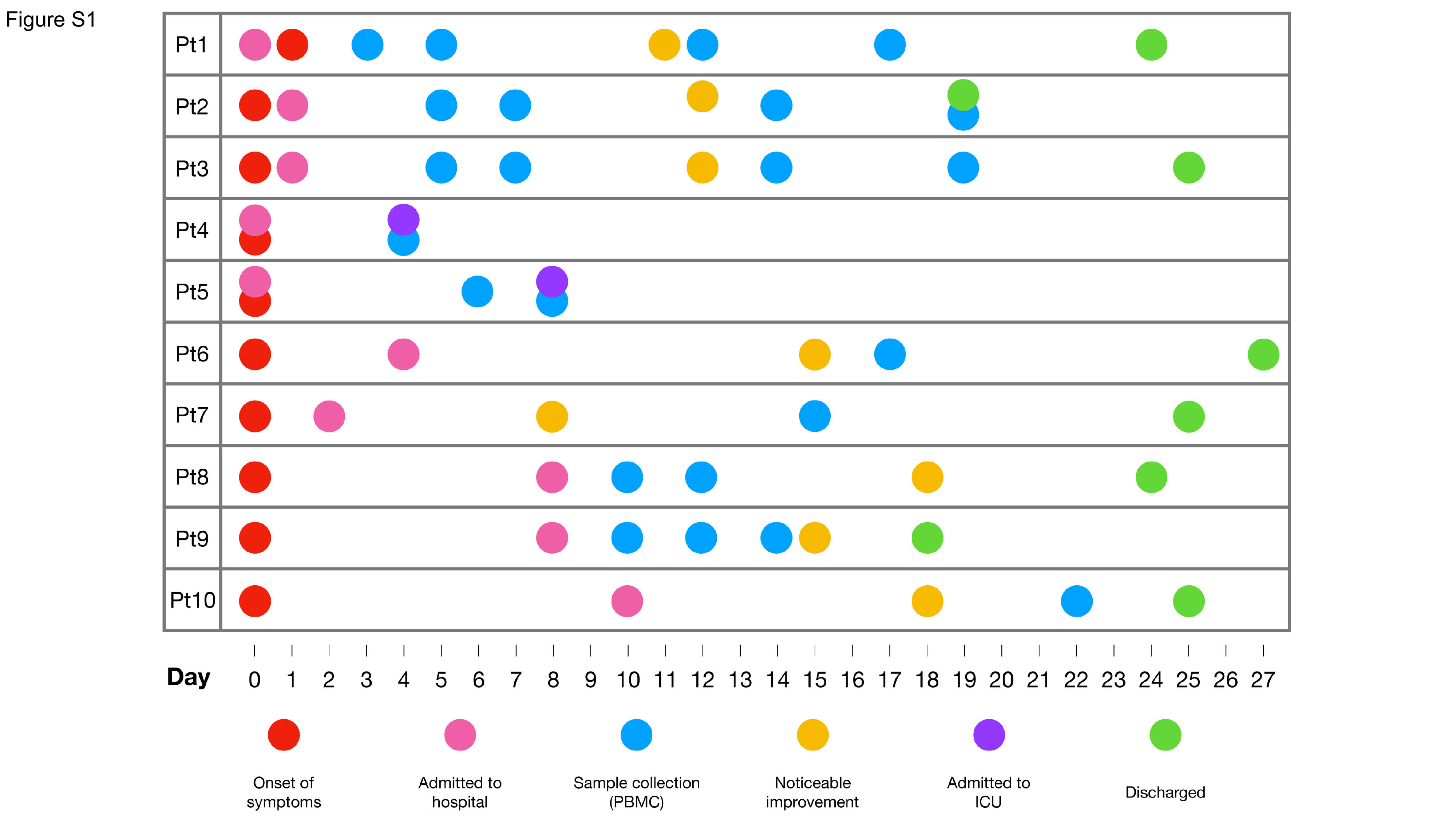

Supplement: Supplementary file 1 [file Image_1.jpeg]
